# The characterization of AD/PART co-pathology in CJD suggests independent pathogenic mechanisms and no cross-seeding between misfolded Aβ and prion proteins

**DOI:** 10.1186/s40478-019-0706-6

**Published:** 2019-04-08

**Authors:** Marcello Rossi, Hideaki Kai, Simone Baiardi, Anna Bartoletti-Stella, Benedetta Carlà, Corrado Zenesini, Sabina Capellari, Tetsuyuki Kitamoto, Piero Parchi

**Affiliations:** 1 Department of Experimental, Diagnostic and Specialty Medicine (DIMES), University of Bologna, Bologna, Italy; 2 IRCCS Istituto delle Scienze Neurologiche di Bologna, Ospedale Bellaria, Via Altura 1/8, 40139 Bologna, Italy; 3 Department of Neurological Sciences, Tohoku University Graduate School of Medicine, Sendai, Japan; 4 Department of Biomedical and Neuromotor Sciences, University of Bologna, Bologna, Italy

**Keywords:** Creutzfeldt-Jakob disease, Prion disease, Prion strain, Aβ-pathology, Tau-pathology, CAA, PART, Neurodegenerative dementia, *APOE*, *PRNP*

## Abstract

Current evidence indicating a role of the human prion protein (PrP) in amyloid-beta (Aβ) formation or a synergistic effect between Aβ and prion pathology remains controversial. Conflicting results also concern the frequency of the association between the two protein misfolding disorders and the issue of whether the apolipoprotein E gene (*APOE*) and the prion protein gene (*PRNP*), the major modifiers of Aβ- and PrP-related pathologies, also have a pathogenic role in other proteinopathies, including tau neurofibrillary degeneration. Here, we thoroughly characterized the Alzheimer’s disease/primary age-related tauopathy (AD/PART) spectrum in a series of 450 cases with definite sporadic or genetic Creutzfeldt-Jakob disease (CJD). Moreover, we analyzed: (i) the effect of variables known to affect CJD pathogenesis and the co-occurring Aβ- and tau-related pathologies; (II) the influence of *APOE* genotype on CJD pathology, and (III) the effect of AD/PART co-pathology on the clinical CJD phenotype. AD/PART characterized 74% of CJD brains, with 53.3% and 8.2% showing low or intermediate-high levels of AD pathology, and 12.4 and 11.8% definite or possible PART. There was no significant correlation between variables affecting CJD (i.e., disease subtype, prion strain, *PRNP* genotype) and those defining the AD/PART spectrum (i.e., ABC score, Thal phase, prevalence of CAA and Braak stage), and no difference in the distribution of *APOE* ε4 and ε2 genotypes among CJD subtypes. Moreover, AD/PART co-pathology did not significantly affect the clinical presentation of typical CJD, except for a tendency to increase the frequency of cognitive symptoms. Altogether, the present results seem to exclude an increased prevalence AD/PART co-pathology in sporadic and genetic CJD, and indicate that largely independent pathogenic mechanisms drive AD/PART and CJD pathology even when they coexist in the same brain.

**Electronic supplementary material:**

The online version of this article (10.1186/s40478-019-0706-6) contains supplementary material, which is available to authorized users.

## Introduction

Creutzfeldt-Jakob disease (CJD) and Alzheimer’s disease (AD) are protein-misfolding disorders characterized by auto-propagation and tissue deposition of protein aggregates leading to progressive neuronal dysfunction and neurodegeneration.

CJD, the most common human prion disease with an estimated incidence of 2 cases per million, comprises several clinical-pathological phenotypes and uniquely occurs in three forms with apparent distinct etiologies [6, 38]. They include sporadic cases of unknown origin, a genetic form linked to mutations in the prion protein gene, *PRNP*, and an infectious form acquired through medical procedures or contaminated food. Current classification of sporadic (s) CJD, the most common form, includes six major disease subtypes that are primarily determined by the combination of the genotype at the polymorphic codon 129 of *PRNP*, encoding for methionine (M) or valine (V), with either one of two types (1 and 2) of misfolded prion protein (PrP^Sc^) that differ in the size (21 and 19 kDa) of their protease-resistant core [55, 56, 58, 59]. Notably, experimental transmissions to syngenic animals demonstrated that prion isolates from 5 of the 6 sCJD subtypes maintain distinctive phenotypic features upon serial passages, supporting the view that they represent different strains of prions [7, 34, 54, 74].

AD, the most common cause of dementia in humans with an estimated prevalence of about 10% in the elderly over 65 years of age [3], involves the misfolding of two proteins. Accordingly, the pathological hallmarks of AD comprise extracellular amyloid plaques consisting of aggregated amyloid beta (Aβ) peptide and intracellular neurofibrillary degeneration (NF), made of paired helical filaments of hyperphosphorylated tau (p-tau) forming neurofibrillary tangles (NFTs), neuropil threads (NThs) and abnormal neurites (Nts).

The amyloid cascade hypothesis, the most widely accepted theory for the molecular sequence of pathological events in AD, postulates that the initial tissue changes in the AD brain involve the aggregation and deposition of the Aβ peptide [33], while the hyperphosphorylation and polymerization of tau into NFTs, NThs, Nts and, ultimately, neuronal loss would occur later. However, the fact that a significant proportion of elderly people accumulates tau earlier or even in the absence of Aβ, and that the first site of brain deposition of the two proteins does not match is apparently in contrast with the primacy of Aβ over tau in AD pathogenesis [9]. To accommodate this apparent discrepancy, a novel neuropathological condition named primary age-related tauopathy (PART) has been recently introduced [18]. The unbound investigation of the pathogenesis and relative risk factors of NF and Aβ pathology allowed by the independent categorization of brains which accumulate NFTs in association with minimal or no Aβ will, hopefully, shed light on the fundamental question of whether PART is indeed a distinct entity unrelated to AD.

Recent experimental evidence suggests that both Aβ and tau aggregates become self-propagating during disease in a prion-like manner [16, 40, 50, 66, 77]. This notion, combined with the frequent coexistence of AD pathology in CJD brains [25, 36], raises the question of the potential cross-seeding between PrP^Sc^, Aβ and tau. Interest into the issue has also been brought up by the finding that the normal cellular prion protein, PrP^C^, can bind different Aβ species, especially oligomers [23, 67], and act as a receptor that internalizes the Aβ aggregates facilitating their neurotoxicity. Nevertheless, current evidence indicating a pathogenic role of PrP in Aβ formation or a synergistic effect between Aβ and prion pathology remains controversial [51, 61, 69, 70].

Conflicting results also concern the frequency of the association between the two pathologies [25, 27, 28, 36, 45]. Moreover, the question of whether the apolipoprotein E (*APOE*) and *PRNP* genotypes, the most significant genetic modifiers of Aβ and PrP^Sc^ pathologies, also affect other proteinopathies, including tau neurofibrillary degeneration, remains largely unsolved [4, 10, 11, 13, 17, 19, 21, 24, 26, 37, 47, 52, 62, 68, 70, 78].

To contribute knowledge on these issues, we thoroughly characterized the AD/PART pathology spectrum in a series of 450 cases with definite CJD, analyzed the effect the CJD molecular subtype, the M/V polymorphism at codon 129 of *PRNP*, and the presence of *PRNP* mutations on the co-occurring neurodegenerative pathology, and evaluated the effect of *APOE* genotype on both CJD and AD/PART pathologies.

## Materials and methods

### Patients and brain tissues

The studied cohort included 450 Italian subjects with definite CJD. All brains were submitted to the Laboratory of Neuropathology at the Institute of Neurological Sciences of Bologna between January 2000 and December 2017. The series comprised consecutive cases referred for diagnosis, except for a few brains lacking frozen tissue or one (e.g., the hippocampus, etc.) or more brain regions required for the application of current criteria for the neuropathological assessment of AD and PART [18, 46, 55], which were excluded.

At autopsy, one half of the brain was immediately frozen and then stored at − 80 °C, while the other half was fixed in formalin. Both frozen and fixed hemispheres were then regionally sampled according to standardized procedures [39, 58].

### Neuropathological analysis

Histopathological examination was performed on 7 μm thick sections of formalin-fixed and paraffin-embedded brain tissue blocks. Sections were systematically taken from neocortical areas (two for each lobe), limbic cortices (cingulate and insular cortices), basal ganglia (anterior and posterior), thalamus (anterior and posterior), hippocampus (anterior and posterior), amygdala, basal forebrain, midbrain, pons, medulla oblongata and cerebellum (vermis and hemisphere with and without dentate nucleus). Evaluations of spongiform change, gliosis, and neuronal loss were performed on hematoxylin and eosin stained sections. The monoclonal antibody 3F4 (1:400, Signet Labs, MA, USA) was used for PrP immunohistochemistry, whereas the antibodies 4G8 (1:5000, Signet Labs, MA, USA) and AT8 (1:100, Innogenetics, Gent, Belgium) were used to assess Aβ and p-tau immunoreactivity, respectively.

### Assessment of amyloid-beta and tau pathology in CJD

**Fig. 1:**
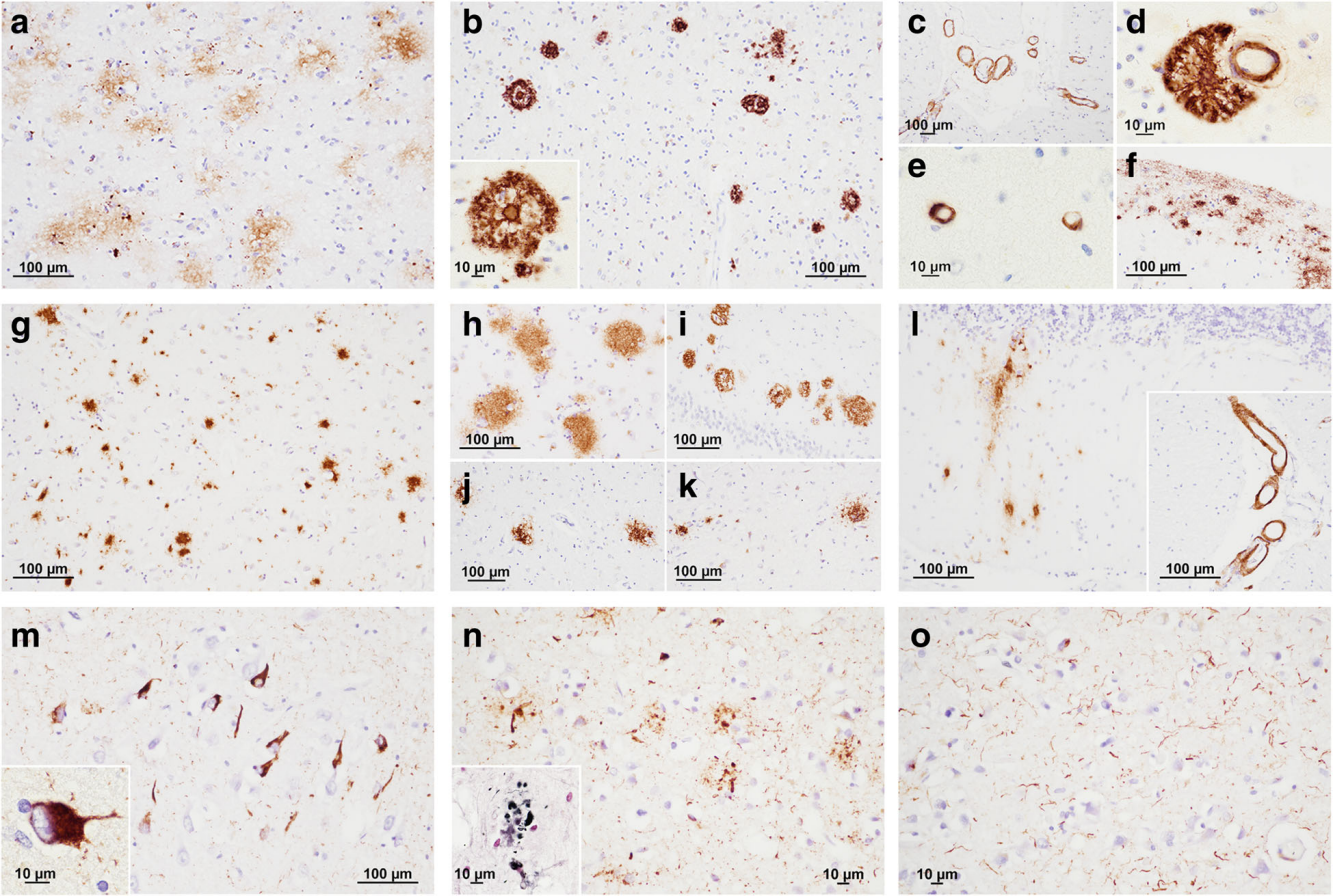
Spectrum of Aβ- and tau-positive lesions in representative cases and brain regions. Aβ + lesions (immunostaining with 4G8 antibody, cerebral neocortex a-f, other regions g-l): **a** Early diffuse Aβ deposits; **b** Aβ core plaques; a higher magnification of a typical core plaque is shown in the lower left box; **c** cerebral amyloid angiopathy (CAA) in medium size parenchymal and leptomeningeal vessels and **e** capillaries; **d** parenchymal CAA with marked perivascular Aβ deposition; **f** Aβ deposits with subpial distribution; **g** dense, coarse Aβ aggregates in the striatum; **h** diffuse Aβ deposits in the amygdala, and **i** the CA1 region of the hippocampus; **j** small focal Aβ deposits in the thalamus, and **k** periaqueductal grey; **l** diffuse Aβ deposits in the molecular layer of the cerebellum; cerebellar leptomeningeal CAA is shown in the lower right box. Tau + lesions (immunostaining with AT8 antibody, m-o): **m** Neurofibrillary tangles (NFT) in the CA1 region of hippocampus of a CJD brain with PART co-pathology; a higher magnification of a globular NFT is shown in the lower left box; **n** dystrophic tau positive neurites contributing to neuritic plaques in the parahippocampal gyrus; a detail of a neuritic plaques (Gallyas silver staining) is shown in the lower left box; **o** numerous neuropil threads in the middle temporal gyrus

Brains were examined independently by two evaluators (PP and HK or MR) for the extent and topographic progression of Aβ deposition (Thal phase), the presence of cerebral amyloid angiopathy (CAA), tau neurofibrillary pathology (Braak stage) and neuritic plaques (CERAD score) as described [1, 2, 8, 39, 43] (Fig. 1). For tau Braak staging, a stage “+” was added for cases showing single or few immunopositive cell bodies (tangles or pre-tangles) in any of the examined brain regions with a distribution pattern that did not fit one of the known tauopathies [1].

Each case was classified according to the level of AD neuropathological change (ABC score) [46], as well as the current diagnostic criteria for PART [18]. To increase the number of cases per group and strengthen the value of statistical tests, we merged some categories defined by the neuropathological scoring systems as follows: for AD neuropathological change (ABC score), we considered Not, Low and Intermediate-High, for Aβ pathology (Thal phase), 0, 1–2, 3 and 4–5, and for tau pathology (Braak stage), 0 − +, I-II and III-VI.

### Molecular genetic analysis

To define the genotype at the polymorphic codon 129 and to identify *PRNP* mutations, the gene open reading frame was sequenced in each case, as previously described [31]. *APOE* analysis was performed through PCR product digestion at 37 °C with the restriction enzyme HhaI (Thermo Fisher Scientific, Waltham MA, USA), and visualized on 3.5% Metaphor agarose gel with GelStar nucleic acid gel stain.

### PrP^Sc^ typing

PrP^Sc^ typing was performed by Western blot using brain homogenates from at least four different brain regions (temporal, parietal, occipital cortices, and thalamus) as described [56, 57].

### CJD classification

All sporadic cases with a definite diagnosis were given a histotype classification according to histopathological features, PrP^Sc^ type, and *PRNP* codon 129 genotypes [55, 58]. Mixed CJD types were merged with the corresponding “pure” subtype based on the main phenotype, as follows: MM(V)1 + 2C merged with MM(V)1, MM2C + 1 with MM2C, MV2K + 2C with MV2K and VV2 + 1 with VV2. Genetic (g) CJD cases were also classified according to the type of mutation and the genotype at codon 129 in the mutated allele (i.e., *PRNP* haplotype).

### Analysis of clinical features

To determine whether the coexisting AD pathology influences the CJD phenotype, we compared clinical features and laboratory findings in two subgroups of 24 patients, selected according to the ABC score (i.e., Not or Low vs. Intermediate or High). We matched the two patient groups according to CJD subtype (all cases were MM(V)1, with an equal proportion of the mixed phenotype MM(V)1 + 2C), gender and age at onset. We compared the following variables: disease duration, symptoms at onset and during the disease course, periodic sharp-wave complexes at EEG (presence or absence), cortical and/or basal ganglia hyperintensities on diffusion-weighted or fluid-attenuated inversion recovery sequences at brain MRI (presence or absence), and abnormal CSF biomarkers, including a positive 14–3-3 protein and/or total-tau protein levels > 1250 pg/ml (presence or absence). Values of CSF Aβ42 and Aβ40 levels and of Aβ42/Aβ40 ratio were also considered, when available.

### Statistical analysis

Statistical analysis was performed using SPSS Statistics version 21 (IBM, Armonk, NY, USA) and SigmaPlot 12.5 (Systat Software Inc). Depending on the data distribution, the results were expressed as mean and standard deviation (SD) or median and interquartile range (IQR).

Differences in age at death or disease duration between CJD with and without AD/PART pathology were evaluated, respectively, with the two-tailed Student’s t-test or the Mann-Whitney U test. Association between categorical variables was assessed by Pearson’s chi-square test. The Spearman’s correlation was used to determine the strength and direction of the relationship between two continuous or ordinal variables. Additionally, multinomial logistic regression analysis was applied to measure the association between molecular and phenotypic subgroups of CJD (independent variables) and levels of AD/PART pathology (dependent variables). All performed multinomial logistic regression analyses were adjusted for age at death and, when stated, also for Thal phase or *APOE* ε4 status.

The most represented categories of the analyzed CJD subgroups and the lowest grades of AD/PART pathology were used as a reference for independent and dependent variables, respectively. Results were expressed as relative risk ratio (RRR) and 95% confidence intervals (CI). A *p*-value < 0.05 was considered statistically significant.

## Results

### Demographic and pathological data of the CJD cohort (Table 1)

The overall mean of age at death and disease duration were, respectively, 68.1 ± 9.0 years and 7.4 ± 11.6 months. As expected, the MM(V)1 subtype comprised the largest group and was associated with the shortest disease duration. Conversely, the MM2T group showed the youngest age at onset and the longest disease duration. Regarding sex, females significantly outnumbered the males in the groups associated with PrP^Sc^ type 2 (Female: type 1 45.6% vs. type 1 + 2 56.4% vs. type 2 62.3%, *p* = 0.010) and the V2 strain (i.e. VV2 and MV2K subtypes combined) (Female: M1 48.3% vs V2 60.6%, *p* = 0.039).

**Table 1:** Demographics of CJD population

	n	Female, n (%)	Age at death (mean years ± SD)	Duration (mean months ± SD)
Total CJD cases	450	234 (52.0)	68.1 ± 9.0	7.4 ± 11.6
Histotype^a^				
MM(V)1	329	159 (48.3)	68.9 ± 8.6	5.5 ± 9.2
VV2	61	36 (59.0)	67.5 ± 9.1	7.4 ± 8.3
MV2K	38	24 (63.1)	65.2 ± 8.3	16.7 ± 12.8
MM2C	13	10 (77.0)	66.8 ± 9.5	16.0 ± 13.5
MM2T	4	2 (50.0)	48.5 ± 14.6	20.0 ± 12.6
VV1	2	1 (50.0)	65.5 ± 0.7	9.8 ± 5.3
VPSPr	2	1 (50.0)	76.5 ± 2.1	21.0 ± 21.2
PrP^Sc^ type^b^				
1	241	110 (45.6)	–	–
1 + 2	101	57 (56.4)	–	–
2	106	66 (62.3)	–	–
Codon 129				
MM	326	164 (50.3)	–	–
MV	60	32 (53.3)	–	–
VV	64	38 (59.4)	–	–

^a^One atypical case was not included

^b^Two VPSPr cases were not included

### Age- and AD-related co-pathologies in CJD

**Fig. 2:**
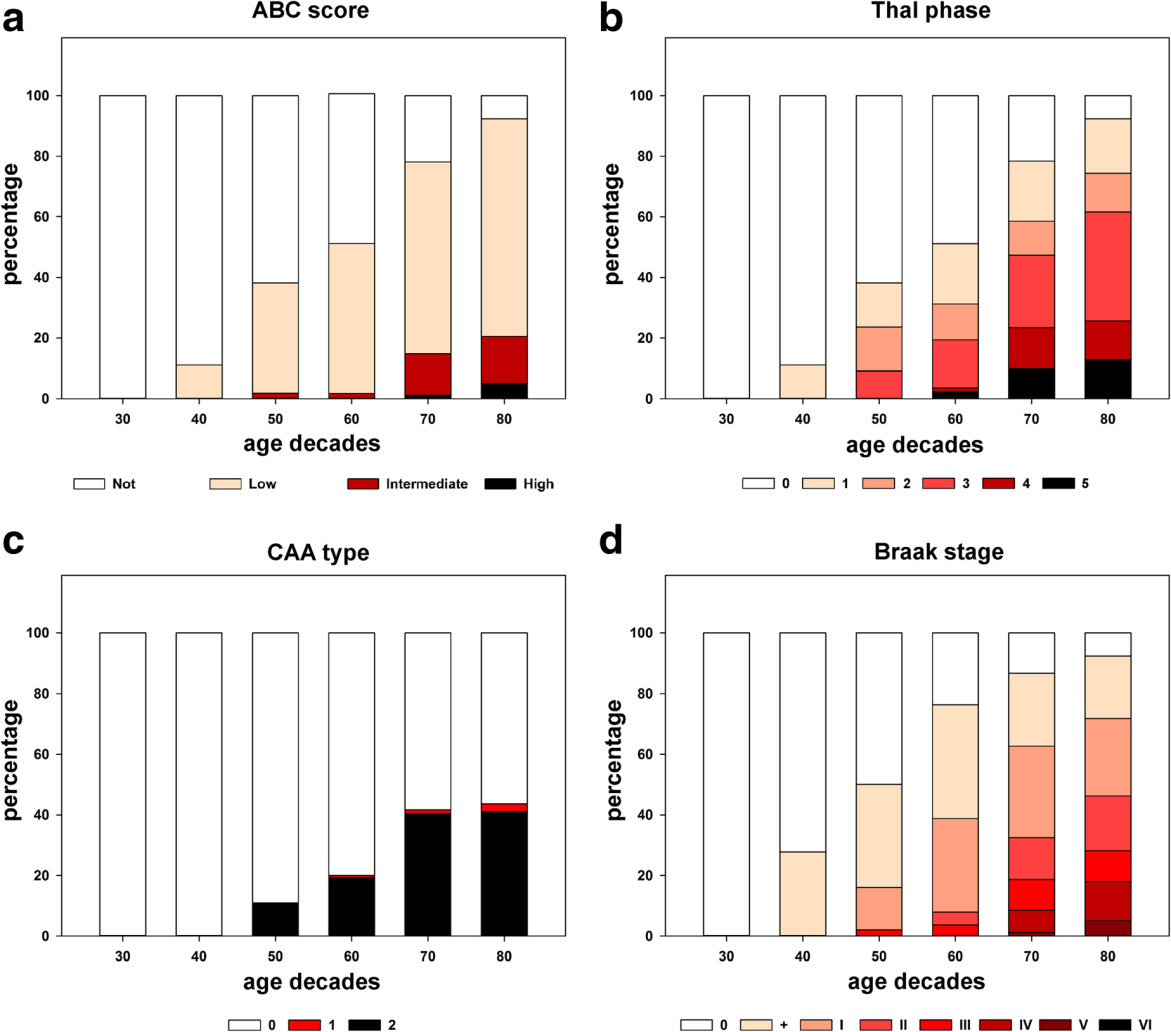
Relationship between PART/AD pathology and age. Levels of AD neuropathological change (**a**), Aβ pathology (**b**), CAA type (type 1 and type 2 were distinguished based on the presence or absence of Aβ deposits in capillaries) (**c**) and tau pathology (**d**) are illustrated in relation to age at death. Color gradation refers to the cases with a given score within each age decade (columns)

A co-occurring pathology belonging the AD/PART spectrum was observed in 333 (74.0%) CJD brains. It was only occasionally seen in subjects in their forties but increased progressively with age (Fig. 2). In the whole population, 240 (53.3%) and 37 (8.2%) of cases, respectively, showed either a low or an intermediate-high level of AD pathology according to the ABC score. Furthermore, 56 (12.4%) and 53 (11.8%) subjects demonstrated neuropathological features compatible with definite and possible PART. Aβ- and tau-related pathologies correlated with age at death, but not with gender or duration of clinical disease (Additional file 1: Table S1 and Additional file 2: Table S2).

### Analysis of the effect of CJD type, prion strain, PrP^Sc^ type and codon 129 genotype on AD/PART co-pathology

The level of AD neuropathological changes, the Aβ phase and the presence of CAA did not show any statistically significant difference between CJD subtypes or strains (Table 2 and Additional file 3: Table S3). Similarly, there was no association between AD pathology and either *PRNP* genotype at codon 129 and PrP^Sc^ type (Additional file 4: Table S4 and Additional file 5: Table S5).

**Table 2:** AD pathology in the different CJD subtypes and strains

	Histotypes, n (%)					Strains, n (%)			
	MM(V)1	VV2	MV2K	Other	p	M1	V2	Other	p
ABC score									
Not	117 (35.6)	29 (47.5)	15 (39.5)	12 (54.5)	0.326	117 (35.6)	44 (44.4)	12 (54.5)	0.178
Low	182 (55.3)	29 (47.5)	21 (55.3)	8 (36.4)		182 (55.3)	50 (50.5)	8 (36.4)	
Intermediate/High	30 (9.1)	3 (4.9)	2 (5.3)	2 (9.1)		30 (9.1)	5 (5.1)	2 (9.1)	
Thal phase									
0	117 (35.6)	29 (47.5)	15 (39.5)	12 (54.5)	0.251	117 (35.6)	44 (44.4)	12 (54.5)	0.087
1–2	99 (30.1)	15 (24.6)	13 (34.2)	6 (27.3)		99 (30.1)	28 (28.3)	6 (27.3)	
3	69 (21.0)	12 (19.7)	8 (21.1)	–		69 (20.1)	20 (20.2)	–	
4–5	44 (13.4)	5 (8.2)	2 (5.3)	4 (18.2)		44 (13.4)	7 (7.1)	4 (18.2)	
CAA									
not CAA	229 (69.6)	47 (77.0)	29 (76.3)	17 (77.3)	0.519	229 (69.6)	76 (76.8)	17 (77.3)	0.330
CAA	100 (30.4)	14 (23.0)	9 (23.7)	5 (22.7)		100 (30.4)	23 (23.2)	5 (22.7)	
Braak stage									
0 − +	163 (49.5)	38 (62.3)	28 (73.7)	13 (59.1)	0.089	163 (49.5)	66 (66.7)	13 (59.1)	0.045
I-II	126 (38.3)	18 (29.5)	8 (21.1)	6 (27.3)		126 (38.3)	26 (26.3)	6 (27.3)	
> III	40 (12.2)	5 (8.2)	2 (5.3)	3 (13.6)		40 (12.2)	7 (7.1)	3 (13.6)	
n	329	61	38	22		329	99	22	

When the analysis was restricted to tau-related pathology the relative percentage of subjects showing a focal NF pathology in the medial temporal lobe (MTL) was significantly lower in the CJD subtypes linked to the V2 strain than in the one linked to the M1 strain [MM(V)1 subtype] (M1 vs V2, stage 0 − + vs stage I-II RRR 0.56 (95% CI, 0.33-0.96) *p* = 0.036) (Table 2, Additional file 3: Table S3). Similarly, likely as a consequence of the absolute predominance the three above-mentioned CJD subtypes within the CJD population, the analysis of tau pathology revealed a significantly lower prevalence of neurofibrillary tau pathology in CJD cases with PrP^Sc^ type 2 than in those with PrP^Sc^ type 1 (type 1 vs type 2, stage 0 − + vs stage I-II RRR 0.55 (95% CI, 0.32-0.95) *p* = 0.033). Notably, the correction for the Thal phase did not affect the correlations between tau pathology in the MTL and the prion strain or the PrP^Sc^ type (Additional file 4: Table S4 and Additional file 5: Table S5).

There were no differences in the distribution of PART between cases stratified according to *PRNP* codon 129 genotype (PART+: MM 25.5% vs MV 18.3% vs VV 23.4%, *p* = 0.480), PrP^Sc^ type (PART+: type 1 26.1% vs type 1 + 2 25.7% vs type 2 18.9%, *p* = 0.322), CJD histotype (PART+: MM1 25.5% vs VV2 23.0% vs MV2K 13.2%, *p* = 0.232) and prion strain (PART+: M1 25.5% vs V2 19.2%, *p* = 0.122).

### AD/PART pathology spectrum in genetic CJD

Previous findings of an excess of AD-related pathological changes in gCJD compared to sCJD [25, 36, 45] led to the suggestion that *PRNP* mutations may favor AD pathology possibly through a cross-seeding mechanism. To address this issue, we compared the relative level of AD pathology between genetic and sporadic CJD. We did not find any statistically significant difference in ABC score, Thal phase, the presence of CAA and tau Braak stage between the two groups. We obtained a similar result when the analysis was limited to the two mutations previously reported to affect Aβ and tau accumulation (i.e., E200K and V210I) (Table 3). As the only exception to this general conclusion, multinomial logistic regression revealed a trend towards an increased percentage of cases with tau or Aβ pathology in gCJD V210I in comparison to sCJD MM(V)1 and, to a lesser extent, gCJD E200K (Additional file 6: Table S6).

**Table 3:** AD neuropahological changes in sCJD and gCJD

	sCJD	gCJD^a^	p	sCJDMM(V)1	V210I	p	E200K^b^	p^c^
ABC score								
Not	144 (37.3)	29 (45.3)	0.481	93 (34.4)	9 (30.0)	0.747	11 (55.0)	0.170
Low	210 (54.4)	30 (46.9)		152 (56.3)	17 (56.7)		8 (40.0)	
Intermediate/High	32 (8.3)	5 (7.8)		25 (9.3)	4 (13.3)		1 (5.0)	
Thal score								
0	144 (37.3)	29 (45.3)	0.577	93 (34.4)	9 (30.0)	0.835	11 (55.0)	0.256
1–2	118 (30.6)	15 (23.4)		84 (31.1)	8 (26.7)		4 (20.0)	
3	76 (19.7)	13 (20.3)		56 (20.7)	8 (26.7)		4 (20.0)	
4–5	48 (12.4)	7 (10.9)		37 (13.7)	5 (16.7)		1 (5.0)	
CAA								
Not CAA	275 (71.2)	47 (73.4)	0.422	187 (69.3)	22 (73.3)	0.409	15 (75.0)	0.398
CAA	111 (28.8)	17 (26.6)		83 (30.7)	8 (26.7)		5 (25.0)	
Braak score								
0 − +	208 (53.9)	34 (53.1)	0.562	133 (49.3)	11 (36.7)	0.377	13 (65.0)	0.328
I-II	133 (34.5)	25 (39.1)		102 (37.8)	15 (50.0)		6 (30.0)	
> III	45 (11.7)	5 (7.8)		35 (13.0)	4 (13.3)		1 (5.0)	
n	386	64	–	270	30	–	20	–
Age at death (years)	68.6 ± 8.9	65.0 ± 9.4	–	69.6 ± 8.3	65.9 ± 8.6	–	63.0 ± 9.1	–

^a^Genetic CJD cases include the following mutations: 30 V210I, 22 E200K, 4 R208H, 2 E219K, 2 V203I, 2 INS (4 repeats), 1 INS (5 repeats), 1 D178N

^b^Two gCJDE200K-129 V showing PrP^Sc^ type 2 were excluded

^c^Pearson’s chi-square test was performed in comparison to sCJDMM(V)1 group

### Influence of *APOE* genotype on CJD and AD pathologies

**Fig. 3:**
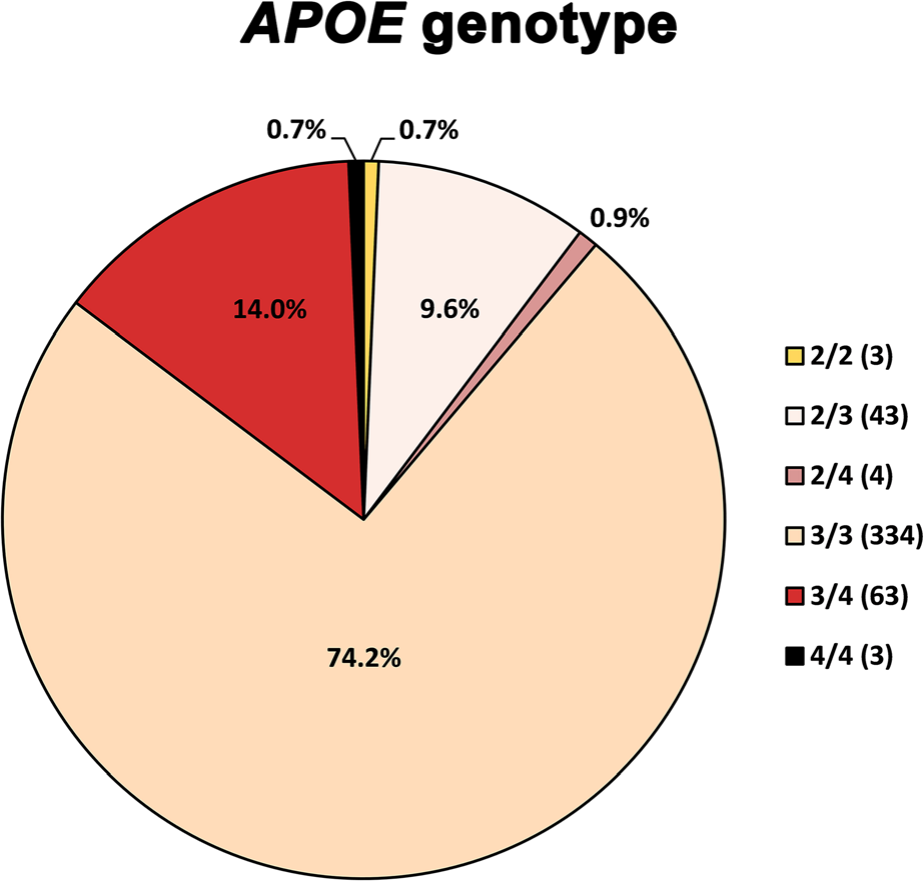
Distribution of *APOE* genotypes in the CJD population. The pie chart describes the relative percentage of each *APOE* genotype (2/2, 2/3, 2/4, 3/3, 3/4, 4/4). The number of cases is specified in brackets

The distribution of *APOE* genotypes in our CJD cohort well matched previous data on the *APOE* allele frequency in both CJD and the general population (Fig. 3) [11, 68]. To further assess the role of *APOE* genotype in prion disease, we performed association analyses based on various histological, biochemical and genetic variables known to affect the CJD pathogenesis and phenotype. The *APOE* ε4 allele distribution (*APOE* ε4+) did not differ between CJD groups classified according to the histotype (MM(V)1 16.4% vs VV2 18.0% vs MV2K 10.5% vs Other 4.1%, *p* = 0.398), PrP^Sc^ type (type 1 18.3% vs type 1 + 2 10.9% vs type 2 14.2%, *p* = 0.202) or *PRNP* codon 129 genotype (ε4+: MM 16.0% vs MV 10.0% vs VV 18.8%, *p* = 0.393) (Additional file 7. Figure S1a, b and c). Furthermore, there was no difference in *APOE* ε4 allelic distribution between genetic and sCJD cases (ε4+: gCJD 20.3% vs sCJD 14.8%, *p* = 0.171) and, in the former group, between patients carrying the E200K and V210I mutations (ε4+: E200K 22.7% vs V210I 23.3%, *p* = 0.830). Finally, we failed to detect any effect of *APOE* ε4 on mean age at onset (ε4–68.12 ± 9.2 vs ε4+ 67.8 ± 8.2, *p* = 0.793), and on disease duration (ε4- median 4.0, IQR 2.5–8.0 vs ε4+ median 4.0, IQR 2.1–6.0, *p* = 0.308) (Additional file 7: Figure S1d).

As expected, the percentage of *APOE* ε4 allele carriers significantly correlated with the level of AD neuropathological changes (ε4+: Not 5.8% vs Low 20.4% vs Intermediate-High 29.7%, *p* < 0.0005), whereas the *APOE* ε2 allele carrier status demonstrated a trend towards a lower grade of AD pathology (ε2+: Not 15.0% vs. Low 9.6% vs. Intermediate-High 2.7%, *p* = 0.056). Tau- and Aβ-related pathologies showed a different association with *APOE*. Indeed, while *APOE* ε4 status and Aβ pathology correlated positively in both parenchyma and vessel walls (Thal phase, ε4+: 0, 5.8% vs 1–2, 15.8% vs 3, 28.1% vs 4–5, 25.5%, *p* < 0.0005; CAA, ε4+: not CAA 10.2% vs CAA 28.9%, *p* < 0.0005), tau pathology did not (ε4+: Braak stage 0, 14.0% vs I-II, 15.2% vs > III, 24.0%, *p* = 0.203) (Table 4). Similarly, the relative protective effect of *APOE* ε2 was confirmed for Aβ pathology but not for tau pathology.

**Table 4:** Influence of *APOE* genotype on AD pathology

	*APOE* ε4 status, n (%)		p
	ε 4-	ε 4+	
ABC score			
Not	163 (42.9)	10 (14.3)	< 0.001
Low	191 (50.3)	49 (70.0)	
Intermediate/ High	26 (6.8)	11 (15.7)	
Thal phase			
0	163 (42.9)	10 (14.3)	< 0.001
1-2	112 (29.5)	21 (30.0)	
3	64 (16.8)	25 (35.7)	
4-5	41 (10.8)	14 (20.0)	
CAA			
not CAA	289 (76.1)	33 (47.1)	< 0.001
CAA	91 (23.9)	37 (52.9)	
Braak stage			
0 − +	208 (54.7)	34 (48.6)	0.203
I-II	134 (35.3)	24 (34.3)	
>III	38 (10.0)	12 (17.1)	
n	380 (84.4)	70 (15.6)	

### Effect of the AD-related pathology on CJD phenotype

**Fig. 4:**
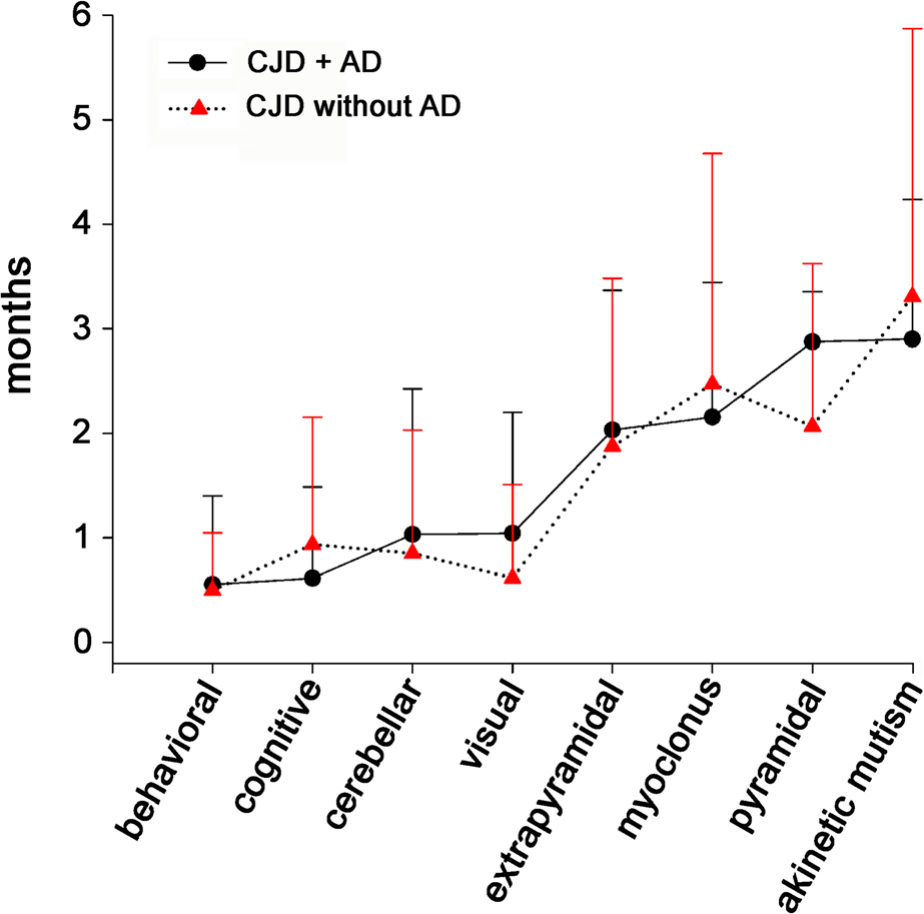
Comparison of clinical course between CJD + AD and CJD without AD. The mean time of appearance (in months) from disease onset is indicated for each group of symptoms/signs. Patients with High or Intermediate AD pathology (CJD + AD group) are compared with a control group with Low or Not AD pathological change, according to the ABC score

To explore the effect of AD-related pathology on CJD clinical and laboratory features, we evaluated two groups of 24 patients significantly differing in the ABC score (24 Intermediate-High, thereafter named as CJD + AD group, and 24 Not-Low, the control group), but matched for CJD subtype, age at disease onset and gender. The mean age in both groups was 76.1 ± 4.6 years and the female: male ratio 1.18 (13 females and 11 males). Clinical manifestations at onset did not differ in the two groups, although cognitive symptoms were slightly more frequent in the CJD + AD group, and cerebellar and visual signs in the control group. No significant differences were detected in the type of symptoms/signs observed, mean time of their appearance from the disease onset (Fig. 4), total disease duration and results of CJD diagnostic investigations (Additional file 8: Table S7). Notably, CSF Aβ42 levels and especially the Aβ42/Aβ40 ratio were significantly lower in the CJD + AD group, which is consistent with the recent demonstration of an inverse correlation between the two CSF biomarkers, especially the latter, and the extent of Aβ deposits in CJD [5, 39].

## Discussion

This is the largest study to date on the characterization of AD/PART co-pathology in a CJD cohort representative of both sporadic and genetic forms and all major disease subtypes. Several previous studies provided evidence of a significant association between CJD and AD in both humans and animal models [25, 36, 79]. These data, combined with others indicating that PrP^C^ acts as a receptor mediating the internalization of Aβ aggregates, facilitating their neurotoxicity, are often taken as evidence of shared pathogenic pathways between the two disorders [35, 65]. Similarly, the recent observation of an unexpected significant Aβ accumulation in the brain of young people affected by iatrogenic CJD, following dura mater graft or treatment with cadaveric-derived human growth hormone (hGH) [12, 32] raised speculations about the possibility of a cross-seeding between PrP^Sc^ and Aβ fostering the formation of Aβ plaques, although the recent demonstration that Aβ deposition is equally high in cadaveric hGH recipients without CJD, suggests that Aβ deposition is triggered by the iatrogenic inoculum independently from the prion pathology [22, 63, 64].

Overall, our results indicate that AD and CJD behave as independent disease processes even when present in the same brain, and strongly argue against shared pathogenic mechanisms between these disorders, which is in line with the conclusions of other investigators based on case-control [17, 60, 80], experimental [62], in-vitro [15], and neuropathological studies [28]. As the only possible exception, we observed a trend towards an increased tau- and Aβ-related pathology in gCJD patients carrying the V210I variant. However, the effect of a shared genetic background related to a common founder, which would fit with the clustering of the mutation in the Italian population, provides an alternative plausible explanation. Furthermore, we detected a significantly lower degree (i.e. lower mean Braak stage) of NF pathology in the CJD subtypes VV2 and MV2K (i.e. those linked to the V2 strain) in comparison to typical CJDMM(V)1, despite the comparable Aβ pathology. The finding may suggest a specific host-genotypic effect on tau pathology, possibly related to *PRNP* codon 129. Given that the CJD MV2K and VV2 subtypes show, on average, more severe pathological changes, including the formation of dot- or stub-like tau deposits in the MTL in comparison to CJDMM(V)1 [39, 56], it is reasonable to believe that the more severe neurodegeneration and the more widespread formation of prion-related tau deposits, possibly causing the sequestration of tau proteins, might have prevented the formation of NF degeneration or made it less visible in such cases. Interestingly, the previous finding that CJDVV2 cases often demonstrate an atypical sub-regional distribution of tau NF degeneration in the hippocampus, and that this occurs more frequently than in other subtypes [35], seems to support this interpretation. Thus, the reduced level of NF pathology in prion strain V2-related subtypes likely reflects the difference in the primary prion pathology. Nevertheless, the possibility that genetic factors linked to codon 129 genotype influence the burden of tau pathology in the MTL in the CJD affected brains cannot be excluded.

The analysis of tau neurofibrillary co-pathology in CJD deserves a further comment. Indeed, at variance with Aβ pathology, tau pathology not only includes the NF degeneration in the cerebral cortex that is spatially linked to senile amyloid plaques (i.e. Braak stages IV-VI), which is unanimously considered AD-related, but also the NF pathology fitting the definition of PART. This adds further complexity since PART and AD pathologies distribute differently in the elderly population, and there is initial evidence indicating that they might be driven by different genetic risk factors [42]. Consequently, they may also differ with respect to their association with CJD. In the present study, we found that PART is equally distributed between cases stratified according to *PRNP* codon 129 genotype, CJD subtype and strain, which support the conclusion of PART and CJD being independent pathological processes.

The strong association between *APOE* ε4 and AD risk [29, 41, 73], and the protective effect of *APOE* ε2 are well established. Our data demonstrating a significant positive association between *APOE* genotype and the level of AD pathology, the Aβ phase and the presence of CAA in the analyzed CJD population are in full agreement with these notions. As in previous studies [20, 44, 71, 72], we also investigated the association between *APOE* genotype and tau pathology (Braak stage) independently from Aβ accumulation (Thal phase). We found no differences in the relative frequency of *APOE* ε4 and ε2 genotypes between tau pathology stages. This result is likely influenced by the fact that, in the large majority of our cases, tau pathology was restricted to the hippocampal region, which as discussed above, might be largely unrelated to AD.

Previous studies also analyzed the influence of *APOE* on CJD, again with discordant results [4, 11, 14, 68, 75, 76]. Recent meta-analysis data involving 1001 CJD patients and 1211 controls suggested an increased risk of developing CJD that is proportional to the number of *APOE* ε4 alleles [78]. Furthermore, proteomic studies pointed to *APOE* as a potential biomarker for prion disease [30, 48] by showing significantly elevated levels of the protein in prion-infected mice [49] and its co-localization with PrP^Sc^ deposits in vivo [53]. In the present study, we systematically analyzed the *APOE* genotype across the whole spectrum of CJD subtypes. We found no differences in the distribution of *APOE* ε4 and ε2 genotypes between CJD subtypes.

Moreover, the presence of a specific *APOE* genotype did not influence the age at onset or the disease duration in our cohort. These observations suggest that *APOE* has no role in the pathogenic mechanism determining the CJD strain and the disease subtype and make the hypothesis of a conformation-specific interaction between PrP^Sc^ and *APOE* unlikely. Finally, the finding that *APOE* is strongly correlated to Aβ pathology but not to CJD pathology further corroborates the view supported by the whole data of the present study, of largely independent pathogenic mechanisms between AD and CJD.

From the clinical point of view, our results indicate that the AD co-pathology does not have a major impact on the clinical presentation of CJDMM(V)1, likely because of the subacute onset and rapid progression of the disease, although it appears to slightly increase the frequency of cognitive symptoms. The possibility that the AD co-pathology might more consistently favor an earlier appearance of cognitive disturbances in other sCJD subtypes, especially those lacking cognitive decline at onset (e.g., VV2 subtype) or manifesting a slower course (e.g. MV2K subtype) remains a possibility.

## Conclusions

By showing that the age-related prevalence and profile of AD/PART co-pathology in CJD is comparable to those described in the normal aging population and demonstrating the lack of any correlation between variables known to affect CJD pathogenesis and those defining the AD/PART pathology spectrum, the results of the present study strongly argue that PART, AD and CJD represent independent disease processes even when present in the same brain.

## Additional files

Additional file 1: **Table S1.** Correlation analyses between AD/PART pathology and sex, age at death, and disease duration. The dependence between factors is analyzed with Spearman’s correlation matrix. (DOCX 16 kb)

Additional file 2: **Table S2.** Correlation analysis between duration of disease and AD/PART pathology. Relative risk ratio (RRR) was calculated by a multinomial logistic regression adjusted by age at death. The lower grades of pathology were chosen as reference categories for the ABC score, Thal phase, CAA and Braak stage. (DOCX 13 kb)

Additional file 3: **Table S3.** Influence of CJD histotype and strain on AD pathology. Relative risk ratio (RRR) was calculated by a multinomial logistic regression adjusted for age at death. For independent variables, MM(V)1 histotype and M1 strain were set as reference groups for histotype and strain analysis, respectively. For dependent variables, the lower grades of pathology were chosen as reference categories for the ABC score, Thal phase, CAA and Braak stage. After controlling for Thal phase, no differences were observed in the Braak stage analysis. (DOCX 17 kb)

Additional file 4: **Table S4.** Level of AD pathology in the CJD groups stratified by *PRNP* codon 129 genotype and PrP^Sc^ type. *The two VPSPr cases were not included. (DOCX 16 kb)

Additional file 5: **Table S5.** Influence of *PRNP* codon 129 and PrP^Sc^ type on AD pathology. Relative risk ratio (RRR) was calculated by a multinomial logistic regression adjusted for age at death. For independent variables, *PRNP* genotype homozygous for methionine (MM) and PrP^Sc^ type 1 were set as reference groups for *PRNP* codon 129 and PrP^Sc^ type analysis, respectively. For dependent variables, the lower grades of pathology were chosen as reference categories for the ABC score, Thal phase, CAA and Braak stage. Further correction for sex and Thal score did not influence the statistically significant negative association between PrP^Sc^ type 2 and Braak score. *Two VPSPr cases were not included. (DOCX 15 kb)

Additional file 6: **Table S6.** Influence of *PRNP* mutations on AD pathology. Relative risk ratio (RRR) was calculated by a multinomial logistic regression adjusted for age at death. For independent variables, sCJDMM(V)1 and gCJD E200K were set as reference groups for the respective analyses. For dependent variables, the lower grades of pathology were chosen as reference categories for the ABC score, Thal phase, CAA and Braak stage. The correction for *APOE* ε4 status did not influence the trend towards a higher AD/PART parhology in gCJD V210I cases. * The analysis was performed on PART negative CJD cases given the higher prevalence of PART positive in gCJD V210I. # The analysis was not carried out (the n was too low). (DOCX 14 kb)

Additional file 7: **Figure S1.** Influence of *APOE* ε4 status on CJD pathology. The distribution of the allele ε4 is not associated with histotype (only the three most represented histotypes are shown: MM1, VV2 and MV2K) (a), PrP^Sc^ type (b) and *PRNP* codon 129 (c). At the same time, the presence of the allele ε4 does not have any effect on age at onset (expressed in years) and disease duration (expressed in months). (TIF 592 kb)

Additional file 8: **Table S7.** Comparison of clinical course between CJD/AD and CJD/notAD groups. The two groups include cases with High or Intermediate (CJD/AD group) and Low or Not (CJD) AD pathological changes, according to the ABC score. *According to Zerr et al. (Zerr I et al. Brain 2009), magnetic resonance imaging (MRI) findings were considered positive when showing (either in diffusion-weighted -DW- or fluid-attenuated -FLAIR- sequences) a hyperintensity in the striatum or in at least two cortical regions; therefore only MRI studies including DW and/or FLAIR sequences were taken into account. ^#^Proteins 14-3-3 and total tau (> 1250 pg/ml). Legend: PSWC, periodic sharp-wave complexes; EEG, electroencephalography; CSF, cerebrospinal fluid. (DOCX 14 kb)

## Abbreviations

AD: Alzheimer’s disease
*APOE*: Apolipoprotein E gene
Aβ: Amyloid-beta
CAA: Cerebral amyloid angiopathy
CI: Confidence intervals
CJD: Creutzfeldt-Jakob disease
CSF: Cerebrospinal fluid
DW: Diffusion-weighted
EEG: Electroencephalography
FLAIR: Fluid-attenuated inversion recovery
gCJD: Genetic Creutzfeldt-Jakob disease
IQR: Interquartile range
M: Methionine
MRI: Magnetic resonance imaging
MTL: Medial temporal lobe
NF: Neurofibrillary degeneration
NFTs: Neurofibrillary tangles
NThs: Neuropil threads
Nts: Abnormal neurites
PART: Primary age-related tauopathy
*PRNP*: Prion protein gene
PrP: Prion protein
PrP^Sc^: PrP scrapie
PSWC: Periodic sharp-wave complexes
p-tau: Hyperphosphorylated tau
RRR: Relative risk ratio
sCJD: Sporadic Creutzfeldt-Jakob disease
SD: Standard deviation
V: Valine
