# Supplementary material for: The characterization of AD/PART co-pathology in CJD suggests independent pathogenic mechanisms and no cross-seeding between misfolded Aβ and prion proteins
Source: Acta Neuropathol Commun. 2019 Apr 8;7:53. doi: 10.1186/s40478-019-0706-6 (PMC6454607; doi:10.1186/s40478-019-0706-6)
Supplement: Supplementary file 1 — Table S1. Correlation analyses between AD/PART pathology and sex, age at death, and disease duration. The dependence between factors is analyzed with Spearman’s correlation matrix. (DOCX 16 kb) [file 40478_2019_706_MOESM1_ESM.docx]

**Additional file 1. Table S1**.

|  |  | **ABC score** | | **Thal phase** | | **CAA** | | **Braak stage** | | **Sex** | | **Age at death** | | **Duration of disease** | |
| --- | --- | --- | --- | --- | --- | --- | --- | --- | --- | --- | --- | --- | --- | --- | --- |
|  |  |  |  |  |  |  |  |  |  |  |  |  |  |  |  |
| **ABC score** | Rho | 1.000 | |  |  |  |  |  |  |  |  |  |  |  |  |
|  | p |  |  |  |  |  |  |  |  |  |  |  |  |  |  |
| **Thal phase** | Rho | 0.909 | | 1.000 | |  |  |  |  |  |  |  |  |  |  |
|  | p | <0.001 | |  |  |  |  |  |  |  |  |  |  |  |  |
| **CAA** | Rho | 0.500 | | 0.548 | | 1.000 | |  |  |  |  |  |  |  |  |
|  | p | <0.001 | | <0.001 | |  |  |  |  |  |  |  |  |  |  |
| **Braak stage** | Rho | 0.396 | | 0.388 | | 0.289 | | 1.000 | |  |  |  |  |  |  |
|  | p | <0.001 | | <0.001 | | <0.001 | |  |  |  |  |  |  |  |  |
| **Sex** | Rho | -0.017 | | 0.014 | | 0.023 | | 0.030 | | 1.000 | |  |  |  |  |
|  | p | 0.716 | | 0.771 | | 0.628 | | 0.530 | |  |  |  |  |  |  |
| **Age at death** | Rho | 0.449 | | 0.480 | | 0.323 | | 0.468 | | -0.030 | | 1.000 | |  |  |
|  | p | <0.001 | | <0.001 | | <0.001 | | <0.001 | | 0.536 | |  |  |  |  |
| **Duration of disease** | Rho | -0.098 | | -0.101 | | -0.066 | | -0.152 | | -0.261 | | -0.181 | | 1.000 | |
|  | p | 0.040 | | 0.034 | | 0.170 | | 0.002 | | <0.001 | | <0.001 | |  |  |
