# Supplementary material for: The characterization of AD/PART co-pathology in CJD suggests independent pathogenic mechanisms and no cross-seeding between misfolded Aβ and prion proteins
Source: Acta Neuropathol Commun. 2019 Apr 8;7:53. doi: 10.1186/s40478-019-0706-6 (PMC6454607; doi:10.1186/s40478-019-0706-6)
Supplement: Supplementary file 2 — Table S2. Correlation analysis between duration of disease and AD/PART pathology. Relative risk ratio (RRR) was calculated by a multinomial logistic regression adjusted by age at death. The lower grades of pathology were chosen as reference categories for the ABC score, Thal phase, CAA and Braak stage. (DOCX 13 kb) [file 40478_2019_706_MOESM2_ESM.docx]

**Additional file 2. Table S2**.

|  | **ABC score** | | **Thal phase** | | **CAA** | | **Braak stage** | |
| --- | --- | --- | --- | --- | --- | --- | --- | --- |
|  |  |  |  |  |  |  |  |  |
| **Duration of disease** | Not as reference | RRR  (95% C.I.) | 0 as reference | RRR  (95% C.I.) | not CAA as reference | RRR  (95% C.I.) | (0-+) as reference | RRR  (95% C.I.) |
|  | Low | 0.983  (0.959-1.007) | 1-2 | 0.984  (0.958-1.011) | with CAA | 0.999  (0.973-1.025) | I-II | 0.971  (0,941-1,003) |
|  | p | 0.160 | p | 0.249 | p | 0.931 | p | 0.076 |
|  | Intermediate/High | 0.985  (0.928-1.046) | 3 | 0.970  (0.930-1.011) | - | - | >III | 0.989  (0.942-1.039) |
|  | p | 0.621 | p | 0.145 | - | - | p | 0.669 |
|  | - | - | 4-5 | 1.003  (0.960-1.048) | - | - | - | - |
|  | - | - | p | 0.890 | - | - | - | - |
