# Supplementary material for: The characterization of AD/PART co-pathology in CJD suggests independent pathogenic mechanisms and no cross-seeding between misfolded Aβ and prion proteins
Source: Acta Neuropathol Commun. 2019 Apr 8;7:53. doi: 10.1186/s40478-019-0706-6 (PMC6454607; doi:10.1186/s40478-019-0706-6)
Supplement: Supplementary file 3 — Table S3. Influence of CJD histotype and strain on AD pathology. Relative risk ratio (RRR) was calculated by a multinomial logistic regression adjusted for age at death. For independent variables, MM(V)1 histotype and M1 strain were set as reference groups for histotype and strain analysis, respectively. For dependent variables, the lower grades of pathology were chosen as reference categories for the ABC score, Thal phase, CAA and Braak stage. After controlling for Thal phase, no differences were observed in the Braak stage analysis. (DOCX 17 kb) [file 40478_2019_706_MOESM3_ESM.docx]

**Additional file 3. Table S3**.

|  | **Histotype** | | | | | | **Strain** | | | |
| --- | --- | --- | --- | --- | --- | --- | --- | --- | --- | --- |
|  | **VV2** | | **MV2K** | | **Other** | | **V2** | | **Other** | |
|  | **RRR (95% CI)** | **p** | **RRR (95% CI)** | **p** | **RRR (95% CI)** | **p** | **RRR (95% CI)** | **p** | **RRR (95% CI)** | **p** |
| **ABC score** |  |  |  |  |  |  |  |  |  |  |
| Not | reference category | | | | | | reference category | | | |
| Low | 0.67 (0.36-1.24) | 0.205 | 1.25 (0.59-2.65) | 0.566 | 0.61 (0.22-1.73) | 0.355 | 0.86 (0.52-1.42) | 0.545 | 0.61 (0.22-1.72) | 0.352 |
| Intermediate/High | 0.42 (0.11-1.58) | 0.199 | 1.00 (0.20-5.09) | 0.996 | 1.09 (0.20-6.05) | 0.921 | 0.58 (0.20-1.69) | 0.319 | 1.09 (0.20-6.02) | 0.923 |
| **Thal phase** |  |  |  |  |  |  |  |  |  |  |
| 0 | reference category | | | | | | reference category | | | |
| 1-2 | 0.63 (0.32-1.29) | 0.209 | 1.28 (0.56-2.90) | 0.555 | 0.80 (0.27-2.38) | 0.802 | 0.84 (0.48-1.48) | 0.555 | 0.80 (0.27-2.37) | 0.686 |
| 3 | 0.73 (0.33-1.61) | 0.439 | 1.32(0.50-30.49) | 0.570 | - | - | 0.92 (0.48-1.76) | 0.801 | - | - |
| 4-5 | 0.47 (0.15-1.42) | 0.181 | 0.68 (0.13-3.46) | 0.640 | 1.52 (0.37-6.32) | 0.560 | 0.54 (0.21-1.41) | 0.211 | 1.53 (0.37-6.31) | 0.560 |
| **CAA** |  |  |  |  |  |  |  |  |  |  |
| 0 | reference category | | | | | | reference category | | | |
| 1 | 0.73 (0.37-1.43) | 0.355 | 0.97 (0.42-2.21) | 0.936 | 0.93 (0.31-2.76) | 0.891 | 0.81 (0.47-1.40) | 0.450 | 0.92 (0.31-2.75) | 0.889 |
| **Braak stage** |  |  |  |  |  |  |  |  |  |  |
| 0-+ | reference category | | | | | | reference category | | | |
| I-II | 0.64 (0.33-1.21) | 0.168 | 0.45 (0.19-1.06) | 0.070 | 0.88 (0,30-2,59) | 0.813 | 0.56 (0.33-0.96) | 0.036 | 0.88 (0.30-2.60) | 0.815 |
| >III | 0.56 (0.19-1.60) | 0.274 | 0.42 (0.09-1.95) | 0.266 | 1.61 (0.38-6.82) | 0.521 | 0.50 (0.20-1.23) | 0.133 | 1.61 (0.38-6.83) | 0.520 |
| **n** | 61 |  | 38 |  | 22 |  | 99 |  | 22 |  |
