# Supplementary material for: The characterization of AD/PART co-pathology in CJD suggests independent pathogenic mechanisms and no cross-seeding between misfolded Aβ and prion proteins
Source: Acta Neuropathol Commun. 2019 Apr 8;7:53. doi: 10.1186/s40478-019-0706-6 (PMC6454607; doi:10.1186/s40478-019-0706-6)
Supplement: Supplementary file 4 — Table S4. Level of AD pathology in the CJD groups stratified by PRNP codon 129 genotype and PrPSc type. *The two VPSPr cases were not included. (DOCX 16 kb) [file 40478_2019_706_MOESM4_ESM.docx]

**Aditional file 4. Table S4**.

|  | ***PRNP* codon 129, n (%)** | | | | **PrP^Sc^ type**^a^**, n (%)** | | | |
| --- | --- | --- | --- | --- | --- | --- | --- | --- |
|  | **MM** | **MV** | **VV** | **p** | **1** | **1+2** | **2** | **p** |
| **ABC score** |  |  |  |  |  |  |  |  |
| Not | 118 (36.2) | 25 (41.7) | 30 (46.9) | 0.500 | 87 (36.1) | 38 (37.6) | 48 (45.3) | 0.384 |
| Low | 181 (55.5) | 29 (48.3) | 30 (46.9) |  | 133 (55.2) | 53 (52.5) | 53 (50.0) |  |
| Intermediate/High | 27 (8.3) | 6 (10.0) | 4 (6.2) |  | 21 (8.7) | 10 (9.9) | 5 (4.7) |  |
| **Thal phase** |  |  |  |  |  |  |  |  |
| 0 | 118 (36.2) | 25 (41.7) | 30 (46.9) | 0.723 | 87 (36.1) | 38 (37.6) | 48 (45.3) | 0.293 |
| 1-2 | 98 (30.1) | 19 (31.7) | 16 (25.0) |  | 75 (31.1) | 28 (27.7) | 30 (28.3) |  |
| 3 | 67 (20.6) | 10 (16.7) | 12 (18.8) |  | 52 (21.6) | 17 (16.8) | 20 (18.9) |  |
| 4-5 | 43 (13.2) | 6 (10.0) | 6 (9.4) |  | 27 (11.2) | 18 (17.8) | 8 (7.5) |  |
| **CAA type** |  |  |  |  |  |  |  |  |
| not CAA | 226 (69.3) | 47 (78.3) | 49 (76.6) | 0.228 | 166 (68.9) | 73 (72.3) | 83 (78.3) | 0.199 |
| CAA | 100 (30.7) | 13 (21.7) | 15 (23.4) |  | 75 (31.1) | 28 (27.7) | 23 (21.7) |  |
| **Braak stage** |  |  |  |  |  |  |  |  |
| 0-+ | 164 (50.3) | 39 (65.0) | 39 (60.9) | 0.144 | 120 (49.8) | 50 (49.5) | 72 (67.9) | 0.028 |
| I-II | 125 (38.3) | 14 (23.3) | 19 (29.7) |  | 92 (38.2) | 39 (38.6) | 26 (24.5) |  |
| >III | 37 (11.3) | 7 (11.7) | 6 (9.4) |  | 29 (12.0) | 12 (11.9) | 8 (7.5) |  |
| **n** | 326 | 60 | 64 |  | 241 | 101 | 106 |  |

^a^ Devoid of two VPSPr cases
