# Supplementary material for: The characterization of AD/PART co-pathology in CJD suggests independent pathogenic mechanisms and no cross-seeding between misfolded Aβ and prion proteins
Source: Acta Neuropathol Commun. 2019 Apr 8;7:53. doi: 10.1186/s40478-019-0706-6 (PMC6454607; doi:10.1186/s40478-019-0706-6)
Supplement: Supplementary file 5 — Table S5. Influence of PRNP codon 129 and PrPSc type on AD pathology. Relative risk ratio (RRR) was calculated by a multinomial logistic regression adjusted for age at death. For independent variables, PRNP genotype homozygous for methionine (MM) and PrPSc type 1 were set as reference groups for PRNP codon 129 and PrPSc type analysis, respectively. For dependent variables, the lower grades of pathology were chosen as reference categories for the ABC score, Thal phase, CAA and Braak stage. Further correction for sex and Thal score did not influence the statistically significant negative association between PrPSc type 2 and Braak score. *Two VPSPr cases were not included. (DOCX 15 kb) [file 40478_2019_706_MOESM5_ESM.docx]

**Additional file 5. Table S5**.

|  | ***PRNP* codon 129** | | | | **PrP^Sc^ type**^a^ | | | |
| --- | --- | --- | --- | --- | --- | --- | --- | --- |
|  | **MV** | | **VV** | | **1+2** | | **2** | |
|  | **RRR (95% CI)** | **p** | **RRR (95% CI)** | **p** | **RRR (95% CI)** | **p** | **RRR (95% CI)** | **p** |
| **ABC score** |  |  |  |  |  |  |  |  |
| Not | reference category | | | | reference category | | | |
| Low | 1.05 (0.56-1.97) | 0.889 | 0.68 (0.37-1.24) | 0.212 | 1.02 (0.60-1.74) | 0.945 | 0.92 (0.55-1.54) | 0.744  0.408 |
| Intermediate/High | 1.90 (0.64-5.66) | 0.252 | 0.62 (0.19-2.06) | 0.437 | 1.32 (0.53-3.31) | 0.549 | 0.63 (0.21-1.89) |  |
| **Thal phase** |  |  |  |  |  |  |  |  |
| 0 | reference category | | | | reference category | | | |
| 1-2 | 1.15 (0.58-2.28) | 0.693 | 0.66 (0.33-1.32) | 0.243 | 0.95 (0.52-1.72) | 0.865 | 0.88 (0.49-1.57) | 0.664 |
| 3 | 1.01 (0.43-2.35) | 0.984 | 0.74 (0.34-1.61) | 0.442 | 0.90 (0.44-1.82) | 0.765 | 0.92 (0.47-1.80) | 0.920 |
| 4-5 | 1.15 (0.39-3.38) | 0.805 | 0.57 (0.20-1.63) | 0.297 | 2.06 (0.92-4.64) | 0.081 | 0.80 (0.31-2.07) | 0.647 |
| **CAA** |  |  |  |  |  |  |  |  |
| 0 | reference category | | | | reference category | | | |
| 1 | 0.79 (0.40-1.58) | 0.505 | 0.73 (0.38-1.41) | 0.354 | 0.91 (0.53-1.57) | 0.742 | 0.74 (0.42-1.30) | 0.298 |
| **Braak stage** |  |  |  |  |  |  |  |  |
| 0-+ | reference category | | | | reference category | | | |
| I-II | 0.59 (0.30-1.18) | 0.138 | 0.67 (0.36-1.25) | 0.209 | 1.13 (0.67-1.92) | 0.647 | 0.55 (0.32-0.95) | 0.033 |
| >III | 1.16 (0.44-3.04) | 0.763 | 0.72 (0.27-1.95) | 0.523 | 1.17 (0.52-2.63) | 0.697 | 0.57 (0.23-1.39) | 0.216 |
| **n** | 60 | | 64 | | 101 | | 106 | |

^a^ Devoid of two VPSPr cases
