# Supplementary material for: The characterization of AD/PART co-pathology in CJD suggests independent pathogenic mechanisms and no cross-seeding between misfolded Aβ and prion proteins
Source: Acta Neuropathol Commun. 2019 Apr 8;7:53. doi: 10.1186/s40478-019-0706-6 (PMC6454607; doi:10.1186/s40478-019-0706-6)
Supplement: Supplementary file 6 — Table S6. Influence of PRNP mutations on AD pathology. Relative risk ratio (RRR) was calculated by a multinomial logistic regression adjusted for age at death. For independent variables, sCJDMM(V)1 and gCJD E200K were set as reference groups for the respective analyses. For dependent variables, the lower grades of pathology were chosen as reference categories for the ABC score, Thal phase, CAA and Braak stage. The correction for APOE ε4 status did not influence the trend towards a higher AD/PART parhology in gCJD V210I cases. * The analysis was performed on PART negative CJD cases given the higher prevalence of PART positive in gCJD V210I. # The analysis was not carried out (the n was too low). (DOCX 14 kb) [file 40478_2019_706_MOESM6_ESM.docx]

**Additional file 6. Table S6**

|  | **sCJD vs gCJD** | | **sCJDMM(V)1 vs V210I** | | **sCJDMM(V)1 vs E200K** | | **E200K vs V210I** | |
| --- | --- | --- | --- | --- | --- | --- | --- | --- |
|  | **RRR (95% CI)** | **p** | **RRR (95% CI)** | **p** | **RRR (95% CI)** | **p** | **RRR (95% CI)** | **p** |
| **ABC score** |  |  |  |  |  |  |  |  |
| Not | reference category | | reference category | | reference category | | reference category | |
| Low | 0.98 (0.53-1.79) | 0.952 | 1.85 (0.72-4.73) | 0.201 | 0.81 (0.28-2.348) | 0.699 | 2.56 (0.68-9.67) | 0.167 |
| Intermediate/High | 1.37 (0.45-4.19) | 0.584 | 3.98 (0.96-16.52) | 0.057 | 0.96 (0.10-9.28) | 0.974 | 4.91 (0.36-67.26) | 0.233 |
| **Thal score** |  |  |  |  |  |  |  |  |
| 0 | reference category | | reference category | | reference category | | reference category | |
| 1-2 | 0.82 (0.40-1.64) | 0.565 | 1.42 (0.50-4.10) | 0.511 | 0.64 (0.18-2.22) | 0.483 | 2.56 (0.54-12.03) | 0.233 |
| 3 | 1.28 (0.59-2.78) | 0.529 | 2.63 (0.86-8.04) | 0.091 | 1.26 (0.33-4.80) | 0.735 | 2.54 (0.52-12.53) | 0.157 |
| 4-5 | 1.38 (0.50-3.79) | 0.537 | 3.90 (1.00-15.17) | 0.050 | 0.67 (0.07-6.44) | 0.727 | # | |
| **CAA** |  |  |  |  |  |  |  |  |
| 0 | reference category | | reference category | | reference category | | reference category | |
| 1 | 1.21 (0.64-2.29) | 0.561 | 1.09 (0.45-2.65) | 0.853 | 1.29 (0.42-3.96) | 0.656 | 0.91 (0.23-3.60) | 0.891 |
| **Braak score** |  |  |  |  |  |  |  |  |
| 0-+ | reference category | | reference category | | reference category | | reference category | |
| I-II | 1.63 (0.88-3.01) | 0.116 | 2.75 (1.12-6.74) | 0.027 | 1.05 (0.35-3.13) | 0.932 | 2.93 (0.73-11.74) | 0.129 |
| >III | 1.16 (0.40-3.36) | 0.785 | 2.74 (0.74-10.14) | 0.132 | 0.67 (0.07-5.85) | 0.714 | 5.06 (0.37-68.64) | 0.223 |
| **n** | 386 vs 64 | | 270 vs 30 | | 270 vs 20 | | 20 vs 30 | |
