# Supplementary figures and images for: The characterization of AD/PART co-pathology in CJD suggests independent pathogenic mechanisms and no cross-seeding between misfolded Aβ and prion proteins
Source: Acta Neuropathol Commun. 2019 Apr 8;7:53. doi: 10.1186/s40478-019-0706-6 (PMC6454607; doi:10.1186/s40478-019-0706-6)

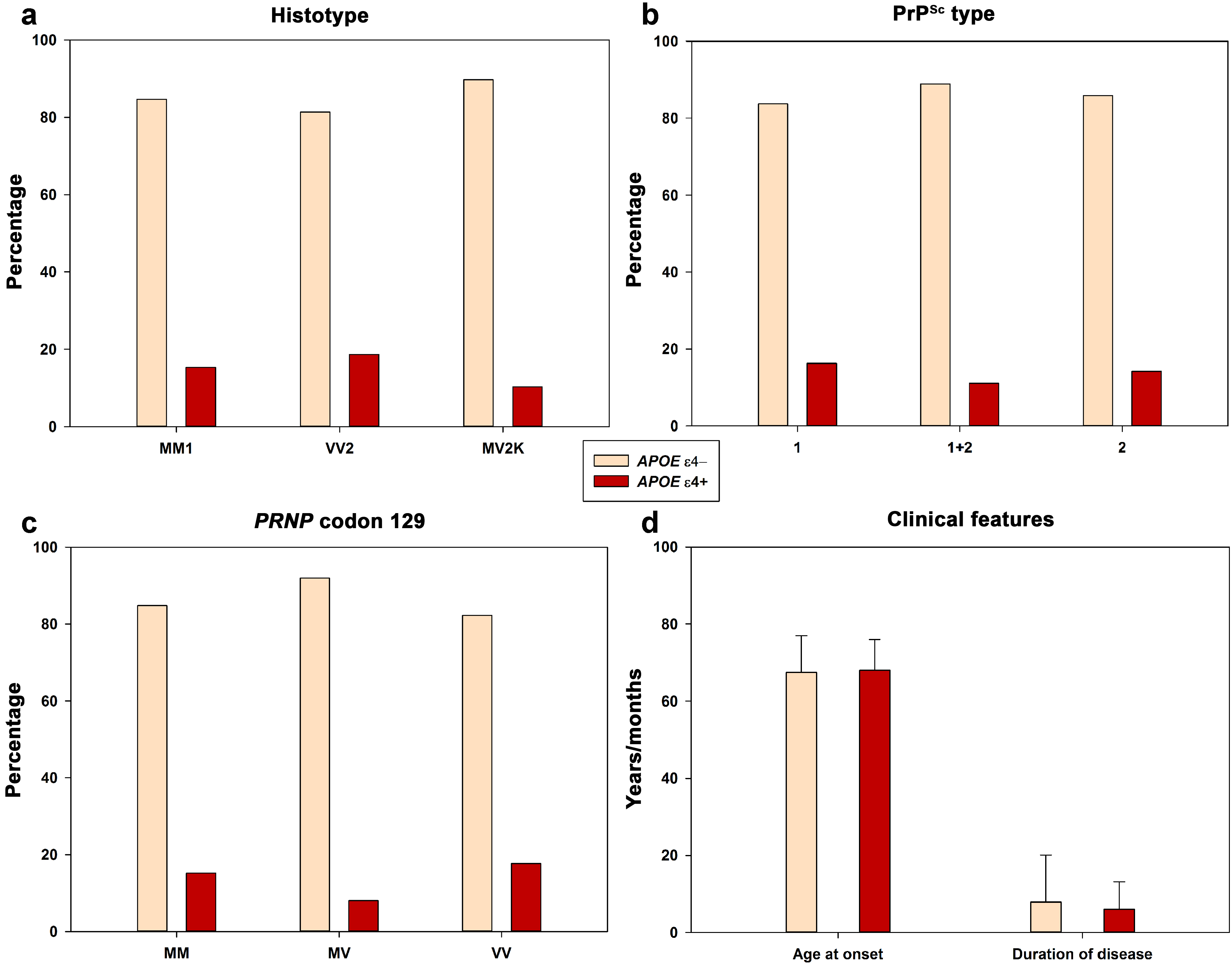

Supplement: Supplementary file 7 — Figure S1. Influence of APOE ε4 status on CJD pathology. The distribution of the allele ε4 is not associated with histotype (only the three most represented histotypes are shown: MM1, VV2 and MV2K) (a), PrPSc type (b) and PRNP codon 129 (c). At the same time, the presence of the allele ε4 does not have any effect on age at onset (expressed in years) and disease duration (expressed in months). (TIF 592 kb) [file 40478_2019_706_MOESM7_ESM.tif]
