# Supplementary material for: The characterization of AD/PART co-pathology in CJD suggests independent pathogenic mechanisms and no cross-seeding between misfolded Aβ and prion proteins
Source: Acta Neuropathol Commun. 2019 Apr 8;7:53. doi: 10.1186/s40478-019-0706-6 (PMC6454607; doi:10.1186/s40478-019-0706-6)
Supplement: Supplementary file 8 — Table S7. Comparison of clinical course between CJD/AD and CJD/notAD groups. The two groups include cases with High or Intermediate (CJD/AD group) and Low or Not (CJD) AD pathological changes, according to the ABC score. *According to Zerr et al. (Zerr I et al. Brain 2009), magnetic resonance imaging (MRI) findings were considered positive when showing (either in diffusion-weighted -DW- or fluid-attenuated -FLAIR- sequences) a hyperintensity in the striatum or in at least two cortical regions; therefore only MRI studies including DW and/or FLAIR sequences were taken into account. #Proteins 14-3-3 and total tau (> 1250 pg/ml). Legend: PSWC, periodic sharp-wave complexes; EEG, electroencephalography; CSF, cerebrospinal fluid. (DOCX 14 kb) [file 40478_2019_706_MOESM8_ESM.docx]

**Addional file 8. Table S7**

|  | **CJD + AD group n=24, (%)** | **Control group n=24, (%)** | **p** |
| --- | --- | --- | --- |
| **Total disease duration** – Mean ± SD | 4.4 ± 2.1 | 4.6 ± 3.0 | 0.828 |
| **Symptom(s) at onset**   - Cognitive - Cerebellar - Visual - Behavioral - Others | 13 (54.2)  6 (25.0)  5 (20.8)  5 (20.8)  0 (0.0) | 8 (33.3)  10 (41.7)  8 (33.3)  5 (20.8)  4 (16.7) | 0.244  0.358  0.516  0.722 |
| **PSWC/tested at EEG** | 17/23 (73.9) | 17/24 (70.8) | 0.928 |
| **Positive/tested brain MRI*** | 7/10 (70.0) | 10/13 (76.9) | 0.917 |
| **Positive/tested surrogate CSF biomarkers of neurodegeneration** | 15/17 (88.2) | 19/20 (95.0) | 0.833 |
| **CSF Aβ42** (pg/ml) | 310  (294-451)  [n=11] | 641  (360-834.5)  [n=9] | 0.015 |
| **CSF Aβ40** (pg/ml) | 6089  (4433.5-6653.5) [n=9] | 5477  (2269.5-8920.5) [n=9] | 0.860 |
| **CSF Aβ42/Aβ40x10** | 0.746  (0.466-0.840) [n=9] | 1.17  (0.940-1.790) [n=8] | 0.003 |
